# NADH/NAD^+^ Redox Imbalance and Diabetic Kidney Disease

**DOI:** 10.3390/biom11050730

**Published:** 2021-05-14

**Authors:** Liang-Jun Yan

**Affiliations:** Department of Pharmaceutical Sciences, College of Pharmacy, University of North Texas Health Science Center, Fort Worth, TX 76107, USA; liang-jun.yan@unthsc.edu; Tel.: +1-817-735-2386; Fax: +1-817-735-2603

**Keywords:** diabetic kidney disease, caloric restriction, NADH/NAD^+^, redox imbalance, mitochondrial homeostasis, mitophagy, oxidative stress

## Abstract

Diabetic kidney disease (DKD) is a common and severe complication of diabetes mellitus. If left untreated, DKD can advance to end stage renal disease that requires either dialysis or kidney replacement. While numerous mechanisms underlie the pathogenesis of DKD, oxidative stress driven by NADH/NAD^+^ redox imbalance and mitochondrial dysfunction have been thought to be the major pathophysiological mechanism of DKD. In this review, the pathways that increase NADH generation and those that decrease NAD^+^ levels are overviewed. This is followed by discussion of the consequences of NADH/NAD^+^ redox imbalance including disruption of mitochondrial homeostasis and function. Approaches that can be applied to counteract DKD are then discussed, which include mitochondria-targeted antioxidants and mimetics of superoxide dismutase, caloric restriction, plant/herbal extracts or their isolated compounds. Finally, the review ends by pointing out that future studies are needed to dissect the role of each pathway involved in NADH-NAD^+^ metabolism so that novel strategies to restore NADH/NAD^+^ redox balance in the diabetic kidney could be designed to combat DKD.

## 1. Introduction

Constituting approximately 0.51% to 1.08% of the body weight, the kidneys are an energy-demanding organ and receive approximately 20–25% of the cardiac output [1,2,3]. Every 24 min, it filters a volume equal to that of whole plasma volume; and every 6 h, it filters a volume equal to that of total body water [3]. Given this workload, the kidney needs a large amount of ATP produced by mitochondria, which, unfortunately, also generate reactive oxygen species (ROS) as metabolic byproducts [4,5,6]. Therefore, the kidney is under constant attack from ROS. Such is indeed the case in diabetic kidney disease (DKD) whereby oxidative stress is elevated and mitochondrial dysfunction is aggravated, leading to renal injury [7,8]. DKD, also known as diabetic nephropathy [9,10,11,12,13], is a common complication of diabetic mellitus, including both type 1 and type 2 diabetes. While type 1 diabetes is caused by lack of insulin due to pancreatic β cell destruction [14,15,16], type 2 diabetes could be caused by insulin resistance or insulin deficiency [17,18,19,20,21,22]. The hallmark of diabetes is a persistent high blood glucose content (hyperglycemia) that can damage a variety of tissues and cells [15,21,22,23]. In the kidney, renal microvascular structures are the major targets of high blood glucose [24,25,26,27]. Additionally, given the facts that the kidney is the organ where mature or active form of vitamin D is made [28,29,30] and erythropoiesis erythropoietin is produced [31,32,33], DKD can also lead to vitamin D deficiency and anemia [34,35,36,37,38,39,40]. Therefore, while there is great understanding of the pathophysiology and progression of DKD, novel and effective treatment approaches are still needed as current therapeutic options remain limited.

While many mechanisms underlie the pathogenesis of DKD including protein kinase C pathway [41,42], hexosamine pathway [43,44], formation of advanced glycation end products [45,46] and the polyol pathway [47,48]; at the molecular level, redox imbalance of NADH/NAD^+^ caused by deranged glucose metabolism [49,50,51] may stand out as a distinct mechanism of diabetic kidney injury [52,53,54,55]. This is because electrons from breakdown of glucose and other nutrients such as fatty acids and amino acids are stored in NADH using NAD^+^ as the electron acceptor [56,57,58]. Therefore, a key feature of diabetes mellitus is oversupply of NADH and under supply of NAD^+^ [48,51,59]. 

## 2. Sources of Elevated NADH in Diabetes

**Figure 1:**
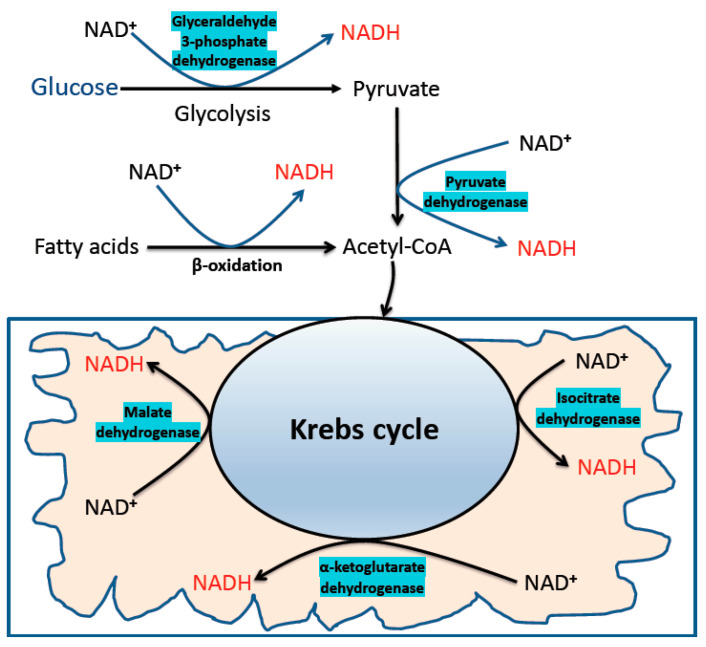
The conventional metabolic pathways that generate NADH from NAD^+^. Shown are the glycolytic pathway, fatty acid oxidation, and the Krebs cycle. These are the major pathways that store electrons in NADH by breaking the chemical bonds in dietary components including glucose, fatty acids. Enzymes involved in direct production of NADH are also indicated in the diagram.

**Figure 2:**
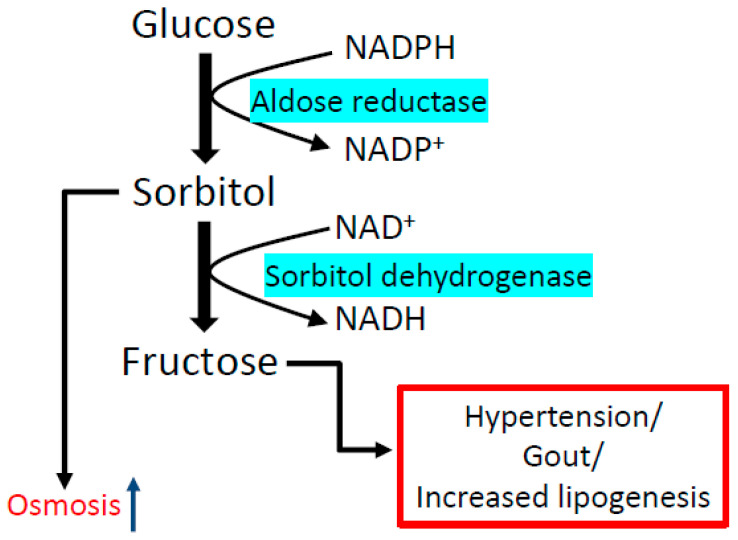
The polyol pathway. This pathway contains two reactions. The first reaction converting glucose to sorbitol is catalyzed by aldose reductase. This enzyme is rate-limiting for the whole pathway. The second reaction converting sorbitol to fructose is catalyzed by sorbitol dehydrogenase. The final products are NADH and fructose, and sorbitol is an intermediate product. Note that NADPH is consumed by aldose reductase in the first reaction. Additionally, accumulation of sorbitol in the kidney could cause osmotic problems for nephrons [63,64].

In addition to the conventional metabolic pathways that extract electrons by breaking the chemical bonds in carbohydrates and fatty acids (Figure 1), other glucose utilization pathways are activated by hyperglycemia [47]. One of these pathways is the polyol pathway [60,61] (Figure 2), which can burn up to 30% of the glucose pool in a diabetic patient [62]. This pathway converts glucose to fructose and also converts NADPH to NADH. There is also an intermediate product known as sorbitol, which could accumulate and impair cellular osmosis in the kidney [63,64]. While the first reaction from glucose to sorbitol is catalyzed by aldose reductase, the second reaction from sorbitol to fructose is catalyzed by sorbitol dehydrogenase. In this pathway, aldose reductase is the rate-limiting enzyme that has a high Km value for glucose [65]. Therefore, numerous studies have focused on aldose reductase as a potential therapeutic target in diabetes [66,67,68,69,70,71]. In particular, attention has been paid to develop small molecule compounds that can inhibit aldose reductase [72,73,74,75,76] to prevent accumulation of sorbitol and fructose and to prevent build-up of NADH, the elevation of which can perturb NADH/NAD^+^ redox balance, initiating reductive stress and oxidative stress. Furthermore, the contribution of the polyol pathway to diabetes development has been demonstrated by the use of aldose reductase animal models whereby lack of aldose reductase prevents the development of diabetes [76]. It should be noted that this NADH/NAD^+^ redox imbalance is also termed as pseudohypoxia in diabetes [77,78] because hypoxia and ischemia often leads to NADH accumulation and NAD^+^ depletion [79,80,81]. It should also be noted that endogenous production of fructose via the polyol pathway has been shown to cause increased fructose and fructose-1-phosphate contents in the kidney, leading to aggravation of DKD [82].

## 3. Pathways of NAD^+^ Consumption in Diabetes

### 3.1. The Poly ADP Ribosylase Pathway

**Figure 3:**
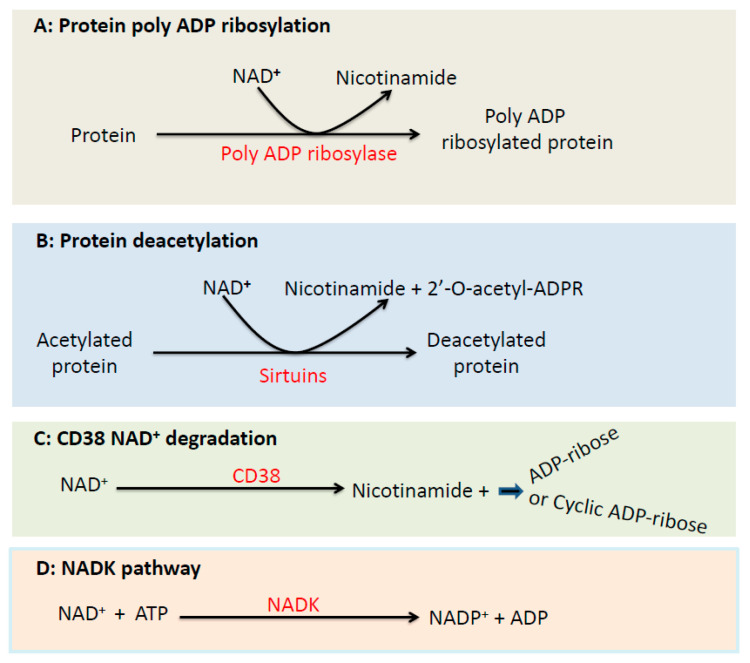
Major pathways that consume NAD^+^. Shown are (**A**) the poly ADP ribosylase reaction; (**B**) the sirtuin-catalyzed deacetylation reaction; (**C**) the CD38 NAD^+^ degradation pathway; (**D**) the NAD kinase pathway converting NAD^+^ to NADP^+^. All the shown pathways or reactions use NAD^+^ as the respective enzyme’s substrate.

While NADH is over-supplied in diabetes, NAD^+^ could be depleted in diabetes. One major pathway utilizing NAD^+^ is the poly ADP ribosylase catalyzed reaction (Figure 3A), which is activated due to DNA damage by ROS in diabetes and uses NAD^+^ as a substrate thereby leading to degradation of NAD^+^ [83,84,85]. The contribution of this pathway to the pathogenesis of diabetes has been confirmed by studies using poly ATP ribosylase deficient mouse, in which lack of the enzyme prevents development of diabetes [86,87], demonstrating the detrimental effects of NAD^+^ depletion in diabetes. 

### 3.2. The Sirtuins Pathway

Another pathway that also consumes NAD^+^ is the sirtuin proteins [88,89] (Figure 3B), which remove acetyl groups from acetylated proteins using NAD^+^ as a substrate. This pathway may play an important role in lowering NAD^+^ levels in early stages of diabetes, but at advanced stages of diabetes, sirtuin protein contents tend to be down regulated [90]. Therefore, it is likely that sirtuin deficiency in advanced stages of diabetes contributes less to NAD^+^ depletion in diabetes.

### 3.3. The CD38 Pathway

CD38 is an NADase that catalyzes the degradation of NAD^+^ [91,92,93] (Figure 3C). This enzyme has been shown to be upregulated in a variety of diseases as well as aging [92,94], leading to decreased content of NAD^+^ that would impair the function of sirtuins and poly ADP ribosylase [95,96]. CD38-driven NAD^+^ deficiency has been shown to be responsible for organ fibrosis and diabetic kidney dysfunction [97]. Conversely, CD38 inhibitors have been shown to mitigate mitochondrial oxidative stress in DKD via restoration of NADH/NAD^+^ redox balance [98].

### 3.4. The NAD Kinase Pathway

**Figure 4:**
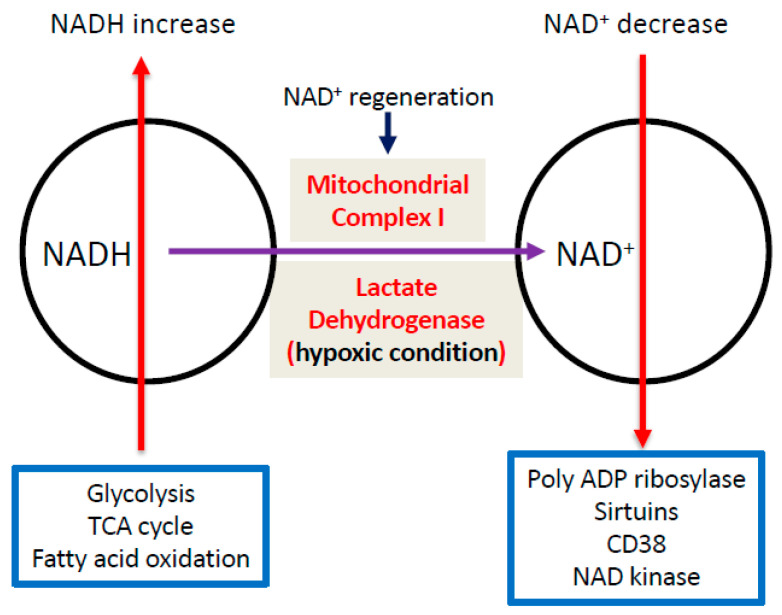
Diagram summarizing the pathways that cause NADH increase and NAD^+^ decrease in the diabetic kidneys. Regeneration of NAD^+^ from NADH by either mitochondrial complex I or lactate dehydrogenase (under hypoxic conditions) is also shown.

NAD kinase (NADK) exists both in the cytosol and in the mitochondria [99,100]. This protein is the sole enzyme responsible for conversion of NAD^+^ to NADP^+^ [101,102] (Figure 3D). Given the key role of NADP^+^ in maintaining the levels of cellular antioxidant glutathione [103,104,105], NADK is an indispensable element in the redox metabolic pathways. Although many studies have been conducted on NADK in a variety of experimental systems, the role of this protein in DKD has yet to be explored. Furthermore, as NADK consumes NAD^+^, how it is involved in maintaining or perturbing NADH/NAD^+^ redox balance in DKD will also need to be investigated. The major pathways causing increase in NADH and decrease in NAD^+^ as well as NAD^+^ regeneration by mitochondrial complex I and lactate dehydrogenase (under hypoxia) are summarized in Figure 4.

## 4. Redox Imbalance-linked Mitochondrial Dysfunction in DKD

One of the major consequences of NADH/NAD^+^ redox imbalance is mitochondrial oxidative stress due to oversupply of NADH to the mitochondrial electron transport chain [47,106,107]. This is caused by electron leakage from the electron transport chain [6,108,109,110,111,112], as it cannot use all of the NADH for ATP production [90,113]. As such, oxygen is partially reduced to form superoxide anion via the electron transport chain, mainly through complexes I, III and IV [114,115]. This mitochondrial superoxide, regardless of the exact site of its generation, is the original source of oxidative stress that can cause oxidative damage to DNA, proteins and lipids [116,117,118,119]. Accumulation of these oxidatively damaged macromolecule adducts can eventually lead to cell death and kidney failure [120,121].

**Figure 5:**
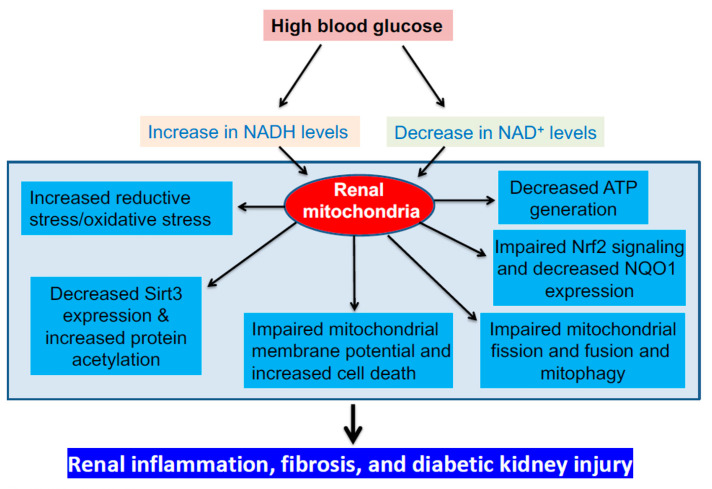
Mitochondrial dysfunction driven by NADH/NAD^+^ redox imbalance and the potential mitochondrial mechanisms underlying pathophysiology of DKD. These mechanisms include increased mitochondrial oxidative damage, decreased ATP production, perturbed mitochondrial membrane potential and deranged mitochondrial homeostasis and impaired sirt3 pathway as well as Nrf2 signaling pathway. The ultimate manifestation of these mitochondrial dysfunctional mechanisms is renal inflammation, fibrosis and diabetic kidney injury.

While NADH/NAD^+^ redox imbalance drives the initial event of superoxide production in mitochondria [47,48,122], other abnormalities of mitochondria could also manifest in DKD, culminating in decreased oxygen consumption and ATP production. As mitochondrion is a dynamic organelle, disruption of its fission and fusion processes [123], also knowns as mitochondrial homeostasis [123,124,125], can also worsen diabetic kidney injury [126]. Indeed, dynamin-related protein 1 (Drp1), well known for its role in regulating mitochondrial fission, has been shown to be upregulated to cause mitochondrial fragmentation in DKD [127,128,129]. Conversely, mitochondrial fusion regulating proteins such as optic atrophy-1 (opa1) and mitochondrial fusion proteins, in particular, mitochondrial fusion protein 2 (Mfn2), have been shown to be down regulated to impair mitochondrial fusion in DKD [130,131,132]. This disruption of mitochondrial homeostasis is linked with redox imbalance and oxidative stress accompanied with impairment of mitochondrial membrane potential and release of apoptosis stimulating factors such as cytochrome c and apoptosis inducing factor (AIF) [133,134,135,136]. These deranged mitochondrial dynamics, if left unattended or uncorrected, would eventually lead to accumulation of damaged mitochondria, which could overwhelm the mitophagy capacity that is regulated by key proteins such as PINK1 and Parkin [137,138,139,140], resulting in cell death and worsened diabetic kidney injury. Therefore, mitochondrial homeostasis and dynamics can also serve as targets for renal therapy in DKD. Figure 5 outlines the potential deleterious mitochondrial consequences of NADH/NAD^+^ redox imbalance implicated in the pathogenesis of DKD.

It is also worth mentioning that a hallmark of the diabetic kidney is hyperfiltration, so that the energy demands of the proximal tubule are greatly increased [141,142,143]. This may temporarily ameliorate the NADH/NAD^+^ redox imbalance as NADH utilization is increased for ATP production. However, as increased NADH consumption means more oxygen consumption and more electron leakage from mitochondria for superoxide production [114,144,145], tubular cells could exhibit increased oxidative stress, which could eventually lead to hyperfiltration linked diabetic nephropathy [53,146]. Nonetheless, whether there is an increased mitochondrial superoxide production linked to hyperfiltration and increased ATP demands remains to be determined.

## 5. Therapeutic Approaches to Counteracting DKD

### 5.1. Superoxide Dismutation and Suppression

There are at least 11 mitochondrial sites that are involved in superoxide generation [147]. Therefore, overall approaches of dismutating superoxide could alleviate DKD [148]. Recently, small molecules that can suppress or inhibit mitochondrial superoxide production have been developed. Typical examples of these small molecules are S1QELs and S3QELs [145,149], which do not interfere with the process of oxidative phosphorylation or ATP production [144]. S1QELs acts at the site I_Q_ of complex I [145,147] while S3QELs acts at the Q site of complex III [150,151]. The usefulness of these suppressors in combating oxidative stress has been tested in certain experimental systems [151,152,153,154]. However, studies of these suppressors in alleviation of DKD have yet to be conducted. It is anticipated that these compounds could attenuate the severity of DKD in diabetic subjects.

### 5.2. Mitochondria-targeted Antioxidants and Superoxide Dismutase (SOD) Mimetics

Antioxidants that go into mitochondria are a class of compounds that can be used to counteract mitochondrial oxidative stress. These are generally purposely synthesized for targeting mitochondria. One well-known compound is mitoQ that has been investigated in a variety of diseases including kidney disease [155,156,157,158,159,160,161,162]. Using type 1 diabetic Akita mouse model, Chacko et al. [163] demonstrated that mitoQ administration over a 12-week period improved tubular and glomerular function in the Akita diabetic mice and decreased urinary albumin content to the level as observed in healthy controls. Moreover, mitoQ-treated Akita mice yielded mitochondria that functioned similar to those isolated from healthy control animals, resulting in attenuation of interstitial fibrosis and glomerular damage. MitoQ could also ameliorate tubular injury by enhancing mitophagy via the Nrf2/PINK1 pathway [164]. In fact, the efficacy of mitoQ in diabetic renal protection is nearly equal to that of angiotensin converting enzyme inhibition [165]. MitoQ could also decrease mitochondrial fragmentation mediated by the JNK signaling pathway in DKD [166]. All these protective effects of mitoQ on DKD can be attributed to its capacity in destroying ROS [167]. It should be noted that while the protective effects of an SOD mimetic tempol has been investigated in DKD [168,169,170,171], the protective effects of other SOD mimetics such as GC4419 [172,173] and EUK189 [174,175,176] are yet to be evaluated in DKD.

### 5.3. Plant and Herb Derived Antioxidants

Numerous plant- or herbal extracts or plant/herb-derived natural products have been tested for their capacity in fighting DKD. A major representative of these extracts is polyphenols that can scavenge ROS [177], leading to correction of redox imbalance and enhancement of mitochondrial function [178,179,180]. Moreover, these plants extracts can also activate the Nrf2 signaling pathway thereby leading to upregulation of the so-called second cellular defense system including antioxidant proteins such as heme oxygenase-1 and NQO1 [181]. As chronic inflammation is implicated in the pathogenesis of DKD, many studies involving plant extracts have also demonstrated their anti-inflammation properties in preclinical DKD [182]. Table 1 shows selected representatives of plant/herb extracts or plant/herb-derived compounds and their redox balanced-related anti-DKD mechanisms.

### 5.4. Caloric Restriction

Caloric restriction (CR) [221,222,223], sometimes also called energy restriction [224,225], is a well-established approach for extending the lifespan of many species. CR can also prolong the health span of many organs including the kidney [226,227,228,229]. As CR has a direct impact on energy supply that involves NADH and NAD^+^, it thus is involved in eliciting antioxidative responses in DKD by restoring redox balance and mitigating diabetic kidney injury [230,231]. Such responses include AMPK activation, autophagy, ROS elimination, Nrf2 signaling pathway activation and enhancement of antioxidative capacity in the kidney [231,232,233,234,235]. In certain studies, exercise has been shown to have a synergistic effect on CR [236,237]. Therefore, CR and exercise may be applied simultaneously to enhance kidney function in diabetes [238,239]. Moreover, intermittent fasting, a different version of CR, has also been demonstrated to prevent progression of DKD via NAD^+^ dependent sirtuin pathway [230]. Additionally, the restriction of single element in a given diet such as iron can also afford renoprotection in diabetes via attenuation of oxidative stress [240]. 

## 6. Magnitude of Redox Imbalance and Progression of DKD

While it is now known that NADH/NAD^+^ redox imbalance is one of the underlying mechanisms of DKD and this redox imbalance drives reductive stress to oxidative stress [47], culminating in renal dysfunction in DKD, whether the magnitude of NADH/NAD^+^ redox imbalance can be associated with the indices of DKD progression has not been established. DKD progression can be determined by the ratio of urinary albumin to urinary creatinine [127] and by estimated glomerular flow rate (eGFR) [241,242], but whether NADH/NAD^+^ ratio would also advance from low to high during DKD progression is unknown at this time and needs to be investigated. It is conceivable that with the progression of DKD quantitated by the above-mentioned parameters, values of the NADH/NAD^+^ ratio would also increase gradually to reflect the severity of DKD. Conversely, the value of the NADH/NAD^+^ ratio should go down upon remission of DKD after treatment. Regardless, this would need to be evaluated using proper animal models that can show clearly an association of the value of NADH/NAD^+^ to progression of DKD until the end stage of renal disease.

## 7. Conclusions

NADH/NAD^+^ redox imbalance, driven by persistent hyperglycemia and oversupply of other nutrients, is the initiator of reductive stress and oxidative stress in DKD [47]. More studies would be needed to dissect the role of each and every player in this cascade of redox imbalance biochemistry mechanism. Complete and comprehensive studies not only will shed insights into the mechanisms of DKD but will also facilitate identification of targets that can be explored for DKD therapy. As indicated in a recent review article by Matoba et al. [243], targeting NADH/NAD^+^ redox imbalance would be a valuable approach for combating DKD. Finally, it should be pointed out that in terms of potential injury caused by redox imbalance, which part of the kidney or what type of cells that sustain the most damage have not been comprehensively evaluated. Therefore, future efforts should be made to assess redox imbalance-induced damage to endothelial cells of the renal vasculature, the podocytes and mesangial cells of the glomerulus and the epithelial cells of the tubule. Additionally, how redox imbalance differs within the tubule should also be measured.

## Figures and Tables

**Table 1:** Selected representatives of plant/herbal extracts/components in DKD from the literature. Experimental models and the major underlying renoprotective mechanisms are also given in the table.

Extracts/Components	Experimental Model	Major Mechanisms	Refs.
Azuki bean extract	* STZ-rat	Autophagy stimulation	[183]
Acacia nilotica	STZ-rat	Antioxidant/anti-hyperglycemia	[184]
Anogeissus acuminate leaf	STZ-rat	Antioxidation	[185]
Broccoli	STZ-rat	Mitigating oxidative damage	[186]
Curcumin	STZ-rat	Inhibiting PKC beta	[187]
Coccinia indica	STZ-rat	Increased antioxidant enzymes	[188]
Coffea arabica pulp	HFD/STZ	Antioxidation upregulation	[189]
Ganoderma lucidum	STZ-rat	TGFβ-1, NFkB	[190]
Garlic extract	STZ-rat	Anti-glycation	[191]
Geraniin	* HFD	Inhibiting oxidative stress	[192]
Ginger extract	STZ-rat	Apoptosis attenuation	[193]
Ginkgo biloba EGB761	HFD/STZ mouse	Mitigating ECM * accumulation	[194]
Berberine	db/db mouse	Mitochondrial fission	[195]
Cupuacu extract	STZ-rat	Mitigating nitrosation	[196]
Anchomanes difformis (leaf)	STZ-rat	Nrf2 activation	[197]
Abelmoschus manihot	HFD/STZ mouse	Autophagy activation	[198]
Hibiscus sabdariffa Linnaeus	STZ-rat	Akt regulating	[199]
Mulberry leaf	HFD/STZ rat	Inhibiting TGF-β1	[200]
Liriope spicata var. prolifera	STZ-rat	Suppressing inflammation	[201]
Nelumbo nucifera leaf	HFD/STZ rat	Antioxidative	[202]
Coreopsis tinctoria nutt	High glucose/HFD/STZ	Anti-fibrotic	[203]
Oil palm	STZ-rat	Attenuating oxidative stress	[204]
Armillariella tabescens	STZ-mouse	Anti-inflammation	[205]
Red ginseng	STZ-rat	Autophagy acceleration	[206]
Paederia foetida leaf	Alloxan-rat	Antioxidative effects	[207]
Tiliacora triandra	HFD/STZ rat	Redox imbalance modulation	[208]
Flavonoids (review article)	Numerous models	Miscellaneous mechanisms	[209]
Grape seed	STZ-rat	Reduce apoptosis	[210]
Grape seed/proanthocyanidins	STZ-rat	Mitigating ER stress	[211]
Grape seed procyanidin B2	db/db mouse	Targeting MFG-E8*	[212]
Grape seed polyphenols	Cell culture	Mitigating oxidative stress	[213]
Catlpol	db/db mouse	Improving lipid metabolism	[214]
Cudrania tricuspidata root	Human kidney cells	Preventing inflammation	[215]
Hyperoside	HFD/STZ mouse	Targeting miR-499-5p?APC	[216]
Phyllanthus niruri leaf	STZ/nicotinamide rat	Anti-fibrosis/apoptosis	[217]
Pomegranate peel extract	STZ-mouse	Nrf2 signaling pathway	[218]
Quercetin	STZ-mouse	Anti-apoptosis/oxidative stress	[219]
Resveratrol	STZ-mouse	Sirt1 activation	[220]

* Abbreviations: HFD, high fat diet; STZ, streptozotocin; ECM, extracellular matrix; MFG-E8, milk fat globule EGF-8. Please note that this table is not meant to exhaust the literature on plant/herbal extracts and DKD.

## Data Availability

Not applicable.
